# Comparison Between Pipeline Embolization Device and Derivo Embolization Device for the Treatment of Unruptured Cerebral Aneurysms: A Single-Center Analysis

**DOI:** 10.3390/jcm15093519

**Published:** 2026-05-05

**Authors:** Weis Naziri, Stefan Daniel Gheorghe, Philipp Dietrich, Michael Kettner, Ruben Mühl-Benninghaus, Umut Yilmaz, Wolfgang Reith, Andreas Simgen

**Affiliations:** 1 Department of Neuroradiology, Philipps University of Marburg, 35043 Marburg, Germany; 2 Department of Neuroradiology, Westpfalz-Klinikum Kaiserslautern, 67655 Kaiserslautern, Germany; sgheorghe@westpfalz-klinikum.de (S.D.G.); asimgen@westpfalz-klinikum.de (A.S.); 3 Department of Neuroradiology, Saarland University Medical Center, 66424 Homburg-Saar, Germany

**Keywords:** flow diverter, intracranial aneurysm, PED, DED

## Abstract

**Background**: The introduction of flow diverters (FDs) has greatly enhanced the treatment of cerebral aneurysms. This study compares two FDs, the Pipeline Embolization Device (PED) and the Derivo Embolization Device (DED), in terms of technical, angiographic and clinical aspects. **Methods**: A total of 103 patients with unruptured aneurysms were treated with the PED (n = 56) and DED (n = 47) between 2012 and 2019. Aneurysm occlusion, procedural complications, occurrence of In-stent stenosis and clinical outcome were evaluated retrospectively. **Results**: Implantation of the flow diverters was technically successful in all patients. There were no significant differences between baseline characteristics and aneurysm morphology. Angiographic follow-up was available with a median short-term follow-up of 3 months and a median long-term follow-up time of 16 months. Adequate aneurysm occlusion at long-term follow-up was substantially but not significantly greater with the DED (95.8%, 45/47) compared to the PED (87.5%, 49/56) (*p* = 0.084). In-stent stenoses were significantly less frequent with the DED (29.8%; 14/47) than with the PED (53.6%, 30/57) at short-term follow-up (*p* = 0.017), although moderate and asymptomatic overall. Thromboembolic or hemorrhagic events occurred in 10.7% (6/56) of cases with the PED and 8.5% (4/47) with the DED (*p* = 0.752). Morbidity rates were similar between devices (PED 3.6% (2/56), DED 2.1% (1/47), *p* = 1.0). There was no procedural mortality. **Conclusions**: Clinical outcomes and complications were comparable between the PED and DED while aneurysm occlusion was considerably greater at long-term follow-up and in-stent stenosis significantly less frequent at short-term follow-up with the DED. The surface-modified design of the DED may contribute to reduced thrombogenicity and early advantages in preventing in-stent stenosis. Further comparative studies are necessary to investigate these findings, particularly comparing surface-modified flow diverters with newer-generation devices featuring true coatings.

## 1. Introduction

Flow diverters (FDs) have been extensively used for the treatment of complex intracranial aneurysms in the past decade. Of all available endovascular treatment options, they represent one of the few methods that allows aneurysm occlusion via the reconstruction of the original vessel wall and thus complete healing of the parent vessel. The first FD that was brought to clinical practice was the Pipeline™ Embolization Device (PED) (Medtronic Inc., Minneapolis, MN, USA) in 2007 [1]. The second generation of FDs entered clinical practice in 2012 and lasted until 2015 and included the Derivo^®^ Embolization Device (DED) (Acandis, Pforzheim, Germany) [2]. In contrast to the PED, the DED was the first surface-modified FD available for clinical practice. The surface finishing of titanium oxides and titanium oxynitrides aims to reduce thrombogenicity [2,3]. Although many studies have shown high rates of aneurysm occlusion and low morbidity rates with the PED and DED [3,4,5,6,7,8,9], data related to the direct comparison of FD types remain scarce. A recently published multicenter study evaluating multiple flow diverter systems, including the PED and DED, demonstrated the favorable safety and efficacy profile of the treatment, characterized by a low incidence of hemorrhagic complications [10]. To date, there is only one comparative study available that analyzed the clinical performance of the PED and DED focusing on aneurysm occlusion, functional outcome and complication rates [5].

The aim of this retrospective study was to share and compare our clinical experiences in the treatment of asymptomatic intracranial aneurysms with the PED and DED with regard to aneurysm occlusions, procedural complications, and clinical outcomes and with particular attention to the occurrence of in-stent stenosis.

## 2. Materials and Methods

### 2.1. Patient Selection

Approval for this retrospective data analysis was granted by the local ethics committee. We reviewed our database and identified patients with unruptured intracranial aneurysms who were treated with PED and DED flow diverters between 2012 and 2019. Aneurysms that were additionally treated with coil embolization were also included. Follow-up evaluations were conducted at two time points using digital subtraction angiography (DSA). Consistent with the current literature and previous studies, the first examination was performed within a short interval of three to six months, whereas the second, longer-term follow-up examination was conducted after six months [11]. Aneurysms additionally treated with coil embolization were also included. 

### 2.2. Devices

The PED (Medtronic/ev3, Irvine, CA, USA) was one of the first FDs to be introduced into clinical practice in 2007 and has been continuously revised. Its body is braided from 48 wires, with a diameter of approximately 30 μm, made of platinum and a cobalt-nickel alloy [1]. The DED (Acandis, Pforzheim, Germany) consists of 48 nitinol composite wires with an inner platinum core and three additional radiopaque markers at each end of the device. In addition, the DED was the first FD with a modified surface. The deep blue colored surface of approximately 50 nm consists of oxides and oxynitrades, aiming for potentially reduced thrombogenicity. Furthermore, in comparison to the PED, the DED has flared ends for optimized wall apposition [2]. 

### 2.3. Antiplatelet Management and Endovascular Procedure

All patients received dual antiplatelet therapy consisting of 100 mg acetylsalicylic acid and 75 mg clopidogrel daily for at least three to five days prior to the intervention. A periprocedural dose of 3000 IU intravenous heparin was administered. Dual antiplatelet therapy was maintained for a minimum of three months following the procedure, after which aspirin was continued indefinitely. Platelet function testing was not performed, as its clinical relevance remains a matter of debate [12]. The procedures were carried out by four neurointerventionalists with five to ten years of experience. All interventions were performed under general anesthesia using a biplane angiography system (Axiom Artis; Siemens Healthcare, Munich, Germany) from 2012–2014, or a biplane flat panel detector angiographic system (Artis Q; Siemens AG, Erlangen, Germany) from 2014–2019. Imaging was performed in posterior-anterior and lateral projection (2D-DSA series) at a rate of 2–4 frames/s, as well as in working position. Sizing of the FD was based on the maximum diameter of the parent vessel into which the FD should be implanted. The device length was chosen based on the width of the aneurysm neck to be covered and the configuration of the parent vessel. All devices were delivered using a 0.027-inch microcatheter (PED: Marksman 27, Medtronic/ev3, Irvine, CA, USA; DED: Neuroslider 27, Acandis, Pforzheim, Germany) via a bi- or triaxial femoral approach using a standard deployment technique. The choice of flow diverter, as well as the decision to deploy multiple devices or to use adjunctive coil embolization, was left to the discretion of the treating neurointerventionalist. Deployment and apposition of the FD to the vessel wall were documented using fluoroscopy. 

### 2.4. Angiographic Evaluation

The angiographic evaluation was performed independently by two experienced neurointerventionalists in consensus. Aneurysm occlusion was assessed using a five point scale previously described in the literature [13,14] as follows: grade 0, no endo-aneurysmal flow changes; grade I, residual filling > 50%; grade II, residual filling < 50%; grade III, near complete occlusion with residual filling at the aneurysm neck; and grade IV, complete occlusion. Aneurysms showing grades III or IV were classified as adequately occluded. In-stent stenosis (ISS) was assessed using the minimum lumen diameter in relation to the vessel diameter at the same location prior to FD implantation. The ISS was rated based on its position (distal, central, or proximal) as well as its grade (mild (<50%), moderate (50–75%), or severe (>75%)). In addition, the braid deformation of both FDs with respect to foreshortening was also investigated. 

### 2.5. Clinical Outcome Evaluation

Clinical outcome was evaluated preinterventionally (baseline), at discharge, at first-follow-up, and at last follow-up using the modified Rankin scale (mRS). Good clinical outcome was classified as mRS 0–2. Treatment-related morbidity was classified as mRS 3–5. Treatment related mortality, classified as mRS 6, was also assessed. 

### 2.6. Statistics

Continuous variables are expressed as mean ± standard deviation. Categorical variables are presented as absolute and relative frequencies, unless stated otherwise. Fisher’s exact and x2 tests were performed for the comparison of categorical variables between the groups. Continuous variables were tested for normal distribution and all groups were compared via one-way ANOVA followed by post hoc analysis including correction of the α-error according to Bonferroni. Statistical significance was accepted at a two-sided *p* value of <0.05. All data analyses were performed using SPSS Statistics Version 25 (IBM Inc., Chicago, IL, USA). 

### 2.7. Ethical Standards

All procedures performed in studies involving human participants or on human tissue were in accordance with the ethical standards of the institutional and/or national research committees and with the 1975 Helsinki declaration and its later amendments or comparable ethical standards. Informed consent was obtained from all individual participants included in the study. Additional written informed consent was obtained from all individual participants or their legal representatives for whom identifying information is included in this article. Ethics committee approval was obtained (identification number 283/19).

## 3. Results

### 3.1. Baseline Characteristics

Among 103 patients with 103 asymptomatic intracranial aneurysms 56 were treated with the PED and 47 were treated with the DED. Neither group had previously undergone endovascular or surgical aneurysm treatment. Patient age differed significantly between the two groups. The population treated with the DED was, on average, six years older than the population treated with the PED (*p* = 0.009, Table 1). The aneurysms treated in this study were predominantly located in the anterior circulation and were mainly located in the various segments of the internal carotid artery (ICA). Baseline aneurysm characteristics were comparable in both groups and are shown in detail in Table 1.

### 3.2. Treatment

FD implantation was technically successful in all cases in both groups. The portion of aneurysms treated with additional coiling was greater in the DED group (19.1%, 9/47) than in the PED group (8.9%, 5/56); however, this difference was not statistically significant (*p* = 0.139). The use of more than one FD in a telescoping technique was necessary in a comparable number of two cases in both groups (PED 3.6% and DED 4.3%, *p* = 0.858). In total, 58 FDs were implanted in the PED group and 49 FDs in the DED group. The distal and proximal overlap of the stent was determined by measuring the distance from the respective end of the stent to the beginning of the aneurysm neck and is given in mm. The PED group showed a distal overlap of 8.8 ± 5.8 mm and a proximal overlap of 13.2 ± 4.8 mm. The DED group showed a distal overlap of 8.6 ± 3.5 mm and a proximal overlap of 14.8 ± 4.4 mm. Overall, the distal and proximal overlap between the PED and DED groups did not show a significant difference (distal *p* = 0.041 and proximal *p* = 0.049). A vessel arising from the base of the aneurysm was detected in a comparable number of two cases (3.6%) in the PED group and in one case (2.1%) in the DED group (*p* = 0.664). The PED group received a slightly longer period of double antiplatelet therapy (DAPT) with Clopidogrel of 8.2 ± 9.9 months compared to the DED group of 7.4 ± 4.0 months; however, this difference was not statistically significant (*p* = 0.298).

### 3.3. Angiographic Follow-Up and Outcome

DSA was available as a follow-up modality for all patients in this study. The first follow-up was performed on the PED group after 3.8 ± 2.1 months and on the DED group after 4.3 ± 2.2 months. For the first follow-up, there was no significant difference between the groups (*p* = 0.144). The last available follow-up for the PED group was after 26.6 ± 24.7 months and for the DED group after 18.1 ± 13.1 months and revealed significantly different (*p* = 0.014) leading to a longer observational period of the PED collective. Aneurysms with Kamran occlusion grades 3 and 4 were classified as adequately occluded. Aneurysms with Kamran occlusion grades 0, 1, and 2 were classified as inadequately occluded (Table 2). At first follow-up adequate aneurysm occlusion was greater in the PED group (80.5%, 45/56) than in the DED group (72.3%, 35/47); however, this difference was not statistically significant (*p* = 0.352). Overall, adequate aneurysm occlusion at final follow-up was greater in the DED group (95.7%, 45/47) than in the PED group (87.5%, 49/56); however, this difference was not statistically significant (*p* = 0.084). 

### 3.4. Clinical Outcome and Complications

All patients in both the PED and the DED groups entered this study with mRS 0. Changes in mRS at discharge were observed in a total of 2 (3.6%) cases in the PED group and in 2 (4.3%) cases in the DED group (*p* = 0.853). In the PED group, one patient suffered a postinterventional mRS of 3 due to an ischemic complication; another patient suffered an mRS of 5 due to a hemorrhagic complication.

One patient treated with a PED exhibited a left-sided paraophthalmic ICA aneurysm. Postinterventionally the patient developed a right-sided minor hemiparesis, a facial mouth weakness and motoric aphasia leading to a National Institutes of Health Stroke Scale Score of 7 (NHISS) and an mRS of 3. Computed tomography angiography and perfusion revealed a perfusion delay in the MCA territory and acute in-stent thrombosis within the PED. Thrombus formation was resolved via performance of aspiration thrombectomy within the PED and administration of Integrilin as an intravenous bolus. Postinterventional Magnetic resonance imaging showed few punctuate embolic infarctions in the MCA territory. The patient was discharged with a mild persisting weakness of the right arm (mRS 1) and was asymptomatic at final follow-up after 12 months.

One patient after treatment of a large right-sided supraophthalmic ICA aneurysm, presented with left-sided hemiplegia, dysarthria and somnolence six hours later, leading to a NHISS of 26 and an mRS of 5. Emergency computer tomography showed a distinct subarachnoid hemorrhage (SAH) and an intraparenchymal hemorrhage (IPH) within the basal ganglia. Recovery from mRS 5 at discharge was only possible to mRS 3 at the last follow-up after 36 months. 

In the DED group, one patient suffered an mRS of 2 and another patient an mRS of 3, both due to an ischemic complication. One patient presented with minor left-sided hemiparesis and hypesthesia after treatment of a supraophthalmic right-sided ICA aneurysm leading to a NHISS of 5 and an mRS of 2. Postinterventional Magnetic resonance imaging showed a small lacunar perforator infarction in the MCA territory. The patient recovered to an mRS of 1 at discharge and was asymptomatic at long-term follow-up after 24 months. Another patient presented with a delayed occlusion of a DED, three weeks after treatment of a left supraophthalmic ICA aneurysm leading to aphasia and right-sided hemiparesis with a NHISS of 10 (NHISS) and an mRS of 3. Computed tomography angiography and Magnetic resonance imaging revealed multiple cortical embolic infarctions in the MCA territory and good contralateral collateralization. Without evidence of a salvageable penumbra, no further revascularization attempt was conducted. The patient was discharged with an mRS 3 and recovered partially at long-term follow-up after 38 months with an mRS of 1.

Minor ischemic events without change in mRS were observed in four cases (7.1%) in the PED group and two cases (4.3%) in the DED group (*p* = 0.686) and detected at postinterventional MRI before discharge. The overall rate of adverse events (ischemic and hemorrhagic) was comparable between groups (PED 10.7% (6/56) and DED 8.5% (4/47), *p* = 0.940).

Incomplete opening with the need for balloon-angioplasty with a compliant remodeling balloon to ensure adequate vessel wall apposition (Eclipse 2; Balt Extrusion, Montmorency, France. Scepter; MicroVention, Tustin, CA, USA) was necessary in one case (1.8%) in the PED group and one case (2.1%) in the DED group (*p* = 1.0).

Distinct foreshortening of the distal portion of the FDs with a resulting incomplete coverage of the aneurysm neck and the indication for implantation of another FD after the first follow-up was observed in five cases (8.9%) in the PED group and one case (1.8%) in the DED group (*p* = 0.142).

Overall, a good clinical outcome (mRS 0–2) was obtained in 54 patients (96.4%) treated with the PED and 46 patients (97.9%) treated with the DED, leading to a treatment-related morbidity rate of 3.6% with the PED and 2.1% with the DED (*p* = 1.0). There was no mortality observed during the complete period of observation in our study.

### 3.5. In-Stent Stenosis 

Overall, we detected an ISS rate of 42.7% (44/103) at short-term and 22.3% (23/103) at long-term follow-up in this study. At short-term follow-up the PED group revealed a significantly greater rate of ISS with 53.6% (30/56) compared to the DED group, with 29.8% (14/47, *p* = 0.017). The ISS frequency ratio balanced out at the long-term follow-up with 25% in the PED group (14/56) and 19.2% in the DED group (9/47, *p* = 0.635). The degree of ISS was comparable in both groups at both follow-up periods and mild in most cases (Table 3). We observed a significant reduction in this ISS grade over time in the PED group of 65% (*p* < 0.001) and in the DED group of 43.9% (*p* = 0.041). In the PED group the ISS was most frequently located in the central portion and in the DED group most frequently located in the distal portion of the implanted devices. In total, we observed one case of severe stenosis in the PED group with 76.3% and one case of moderate stenosis in the DED group with 53.1%. None of the patients presented with neurological symptoms that could be associated with the observed ISS.

## 4. Discussion

The literature on flow diverters is rich and evolving, with ongoing research focused on refining patient selection criteria, improving device technology, and understanding long-term outcomes and complications. This study compared aneurysm occlusion rates, procedural complications, clinical outcomes, and in-stent stenosis between patients treated with the PED and DED. While Zaeske et al. in 2021 [5] and Goertz et al. in 2026 [10] compared aneurysm occlusion rates, functional outcomes, and complications for both devices, to our knowledge, this represents the first investigation specifically examining in-stent stenosis occurrence between these flow diverters.

The follow-up interval after flow diversion is critical for evaluation, as complete aneurysm occlusion can take months to years [4]. While 12 months is widely accepted for long-term follow-up [13], results beyond 3–5 years remain rare due to pending data and continuous device development. With a median follow-up of 21.6 months, this study provides robust long-term data, including 28 patients with over 3 years and 11 patients with over 5 years of follow-up. Aneurysms treated with DED had significantly shorter second follow-up (*p* = 0.017), as this device was the newest utilized, having been available only since 2015. This limitation should be considered when evaluating aneurysm occlusion rates and in-stent stenosis occurrence in longer-term follow-up.

The Kamran five-point scale effectively assessed occlusion after flow diverter treatment of saccular and fusiform aneurysms [14]. Overall, adequate aneurysm occlusion was achieved in 91.3% of cases at last follow-up: 87.5% for the PED and 95.7% for the DED. These results align with long-term occlusion rates reported in other studies [4,5,15]. Occlusion rates between devices approached statistical significance (*p* = 0.084). However, the median second follow-up in the PED group occurred approximately eight months later than in the DED group. Therefore, it remains possible that occlusion rates could have reached statistical significance if assessed at comparable follow-up intervals. Six aneurysms treated with the PED showed insufficient occlusion: three due to incomplete coverage from foreshortening (grade 0), and three involving branch arteries emerging from the aneurysm. While complete occlusion is unlikely in branch-incorporating aneurysms [16], partial occlusion (grade 2) was achievable with flow diverter treatment.

Overall, good clinical outcomes (mRS 0–2) were achieved in 96.4% of PED-treated and 97.9% of DED-treated patients, with treatment-related morbidity rates of 3.6% and 2.1%, respectively (*p* = 1.0), and no procedural mortality. These findings align with the existing literature [5,6]. One hemorrhagic complication occurred: a patient experienced SAH and IPH 12 hours after PED treatment. Brinjikji et al. reported SAH and IPH incidences of 4% and 3%, respectively [17]. Proposed mechanisms for delayed SAH include altered intra-aneurysmal flow conditions and proteolytic enzymes from intra-aneurysmal thrombus. Rouchaud et al. demonstrated that up to 50% of delayed SAH cases occur in giant aneurysms, and previously coiled aneurysms rupture in up to 20% of cases [18]. The mechanism of IPH remains uncertain, with hemorrhagic transformation after ischemia, hemodynamic changes, and DAPT as potential causes [19]. Ischemic complications occurred in 9% of cases (six minor, two major strokes) without significant difference between devices. The overall adverse event rate (PED 10.7%, DED 8.5%) remained within the reported literature ranges of 16% for the PED and 9.5% for the DED [20,21].

Consistent with the existing literature, foreshortening occurred more frequently with the PED (8.9%, n = 5) [22] than the DED (1.8%, n = 1), resulting in insufficient aneurysm occlusion due to incomplete coverage. Foreshortening and migration are recognized complications with reported prevalence rates of 2.2–4.9%, potentially increasing mortality. The deployment technique and device undersizing are contributing factors [23].

Intimal hyperplasia causing transient in-stent stenosis (ISS) is a common finding after flow diverter treatment, typically occurring at short-term follow-up, declining over time, and rarely causing neurological symptoms [24]. The observed stenoses in our study were all moderate, with a frequency of 56.7%, consistent with reported rates (30–57%) [24,25]. Recent studies suggest that ISS occurs more frequently in younger, predominantly female patients, with highly braided flow diverters, and in ruptured aneurysms [26]. It is noteworthy that patients in the DED group were older compared to those in the PED group (mean age PED: 51.2 ± 12.2 years vs. DED: 57.1 ± 11.3 years, *p* = 0.09). Treatment indications were determined by the interventionalist without specific age-based selection. In the absence of a statistically significant difference, no further age-adjusted analyses were performed. The baseline characteristics of this cohort, with mean ages and predominant anterior circulation location within ICA segments, are consistent with the current literature. Notably, higher age, particularly in patients aged 65–74 years and ≥75 years—who exhibit increased risk of residual aneurysm filling at 6 months [27]—appears to be protective against ISS development [28]. ISS showed no significant differences between devices at first and last follow-ups (*p* = 0.760 and *p* = 0.384, respectively). The DED demonstrated significantly lower ISS frequency at first follow-up compared to the PED (*p* = 0.017). However, this was not confirmed at long-term follow-up, where rates were comparable (DED 9/47, PED 14/56); this is likely attributable to DED’s significantly shorter follow-up period (*p* = 0.014). The lower initial ISS occurrence with DED could potentially be explained by its modified surface designed to reduce thrombogenicity.

Overall, the surface-modified DED group demonstrated markedly reduced ISS rates within a comparable short-term follow-up period, while achieving similar aneurysm occlusion rates. Despite a shorter interval to the second follow-up assessment, the DED group tended to show higher aneurysm occlusion rates and lower ISS rates. Future studies should focus on comparisons with true coating flow diverters, whose surface modifications actively interact with the coagulation cascade. Beyond potential differences in aneurysm occlusion and ISS rates, variations in thromboembolic events, antiplatelet regimens, and stent deformation characteristics may also be hypothesized.

## 5. Conclusions

This study demonstrates that both the PED and DED achieve favorable long-term outcomes, with adequate aneurysm occlusion rates of 87.5% and 95.7%, respectively, and excellent clinical outcomes (mRS 0–2) in over 96% of cases. Complication rates were comparable between devices and consistent with the published literature, though foreshortening occurred more frequently with the PED. Although the study was conducted using older-generation devices, it demonstrated overall acceptable safety and efficacy outcomes compared with newer devices. In particular, the surface modified DED showed a clear advantage over the PED regarding the occurrence of in-stent stenosis at short-term follow-up, although this difference diminished over time, likely due to the shorter follow-up period available for the DED. The modified surface of the DED, designed to reduce thrombogenicity, may contribute to this early benefit. Further studies are warranted to investigate this finding, especially in comparison with newer FDs with true coatings – for example, the Derivo 2 Heal device with a fibrin and heparin-based coating and phenox devices with a hydrophilic polymer coating. Long-term follow-up data with these new FD systems will be essential to determine whether early advantages in ISS reduction translate into sustained clinical benefits and improved patient outcomes.

## Figures and Tables

**Table 1:** Demographics, aneurysm location and morphology.

	PED (56)	DED (47)	*p*-Value
Mean age, years	51.2 ± 12.2	57.1 ± 11.3	0.009
Sex			
Female	44 (78.6%)	37 (78.7%)	0.985
Male	12 (21.4%)	10 (21.3%)	0.985
Aneurysm location			
Anterior circulation	49 (87.5%)	45 (95.7%)	0.140
Supraophthalmic ICA	15 (26.8%)	10 (21.3%)	
Paraophthalmic ICA	18 (32.1%)	22 (46.8%)	
Infraophthalmic ICA	16 (28.6%)	13 (27.7%)	
Posterior circulation	7 (12.5%)	2 (4.3%)	
Vertebral artery (VA)	7 (12.5%)	2 (4.3%)	0.140
Aneurysm morphology			
Aneurysm size [mm]	5.4 ± 3.6	5.1 ± 2.7	0.579
Aneurysm neck [mm]	3.7 ± 2.2	3.9 ± 1.7	0.654
Dome-to-neck ratio	3.2 ± 1.3	3.5 ± 1.4	0.917
Saccular	51 (91.1%)	43 (91.5%)	0.940
Fusiform	5 (8.9%)	4 (8.5%)	0.940

**Table 2:** Five-point grading scheme of aneurysm occlusion.

Occlusion ([14])	PED (56)	DED (47)	*p*-Value
First Follow-up			0.352
Grade 0	3	0	
Grade 1	4	5	
Grade 2	4	8	
Grade 3	4	1	
Grade 4	41	33	
Last Follow-up			0.084
Grade 0	2	0	
Grade 1	1	0	
Grade 2	4	2	
Grade 3	1	3	
Grade 4	48	42	

**Table 3:** In-Stent Stenosis.

ISS	PED (56)	DED (47)	*p*-Value
First Follow-up	30	14	0.017
Last Follow-up	14	9	0.635
First Follow-up	32.6 ± 17.5	27.8 ± 12.7	0.760
Last Follow-up	11.4 ± 13.3	15.6 ± 14.8	0.384
Distal	13	7	
Central	15	3	
Proximal	2	4	

## Data Availability

The data that support the findings of this study are available from the corresponding author on reasonable request.

## Abbreviations

The following abbreviations are used in this manuscript:

DAPT	dual antiplatelet therapyMultidisciplinary Digital Publishing Institute
DED	Derivo^®^ Embolization Device
DSA	digital subtraction angiography
FD	flow diverter
ICA	internal carotid artery
IPH	intraparenchymal hemorrhage
ISS	in-stent stenosis
mRS	modified Rankin scale
PED	Pipeline™ Embolization Device
SAH	subarachnoid hemorrhage
NHISS	National Institutes of Health Stroke Scale Score
